# P-1945. Initial Empirical Echinocandin Therapy for Candidemia and the Risk of Ocular Candidiasis: A Retrospective Study

**DOI:** 10.1093/ofid/ofaf695.2113

**Published:** 2026-01-11

**Authors:** Seung Hoo Lee, Jinyoung Yang, Jae-Hoon Ko, Sun Young Cho, Cheol-In Kang, Doo Ryeon Chung, Kyong Ran Peck, Kyungmin Huh

**Affiliations:** Samsung Medical Center, Sungkyunkwan University School of Medicine, Seoul (Gangnam-gu), Seoul-t'ukpyolsi, Republic of Korea; Samsung Medical Center, Seoul, Seoul-t'ukpyolsi, Republic of Korea; Samsung Medical Center, Seoul, Seoul-t'ukpyolsi, Republic of Korea; Samsung Medical Center, Seoul, Korea, Seoul, Seoul-t'ukpyolsi, Republic of Korea; Samsung Medical Center, Seoul, Seoul-t'ukpyolsi, Republic of Korea; samsung medical center, Seoul, Seoul-t'ukpyolsi, Republic of Korea; Samsung Medical Center, Seoul, Seoul-t'ukpyolsi, Republic of Korea; samsung medical center, Seoul, Seoul-t'ukpyolsi, Republic of Korea

## Abstract

**Background:**

Ocular candidiasis (OC) is a complication of candidemia. While echinocandins are recommended as first-line therapy for candidemia, their poor ocular penetration raises concern. This study evaluated whether initial echinocandin use increases the risk of OC.

**Figure:**
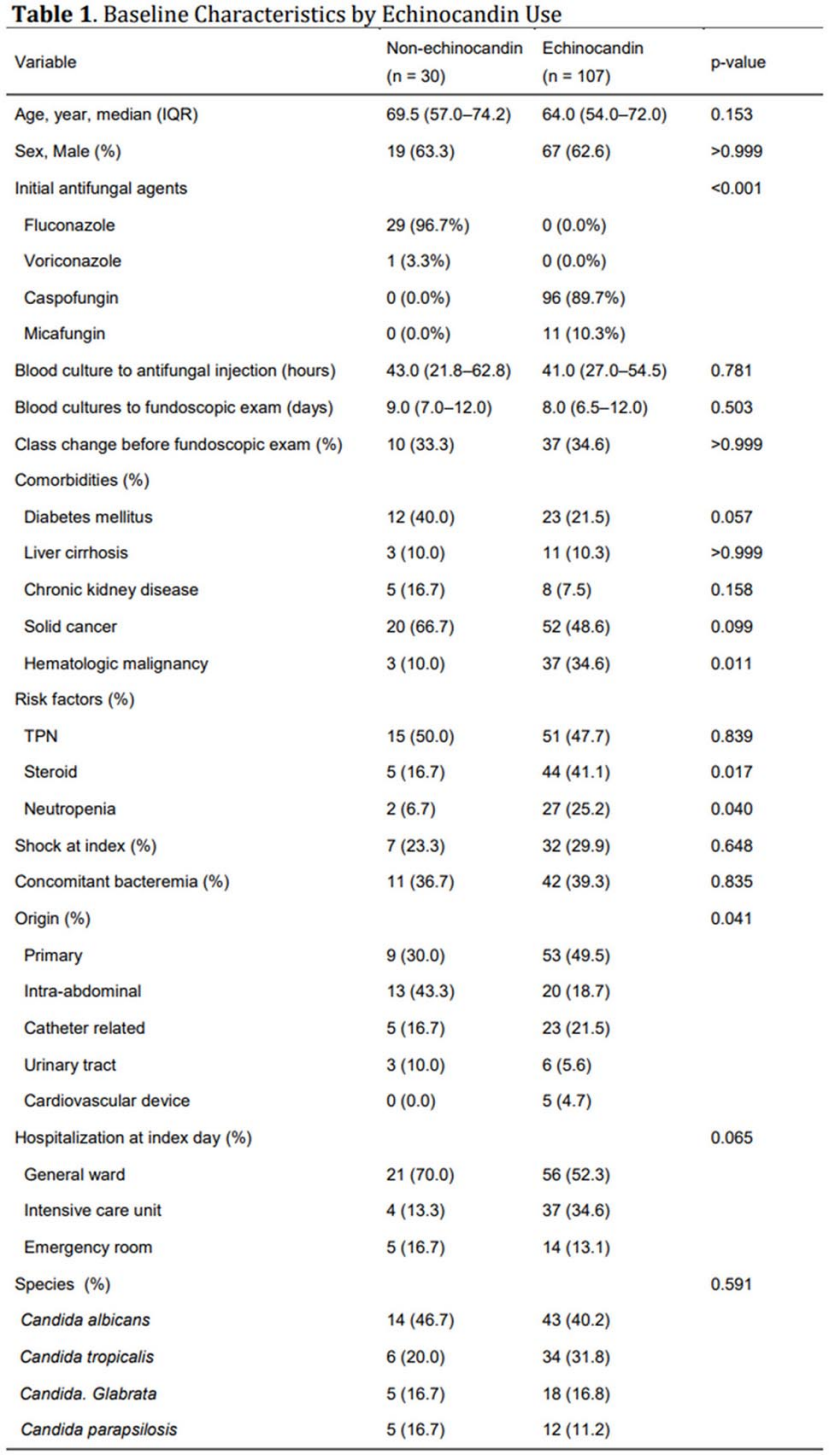

**Figure:**
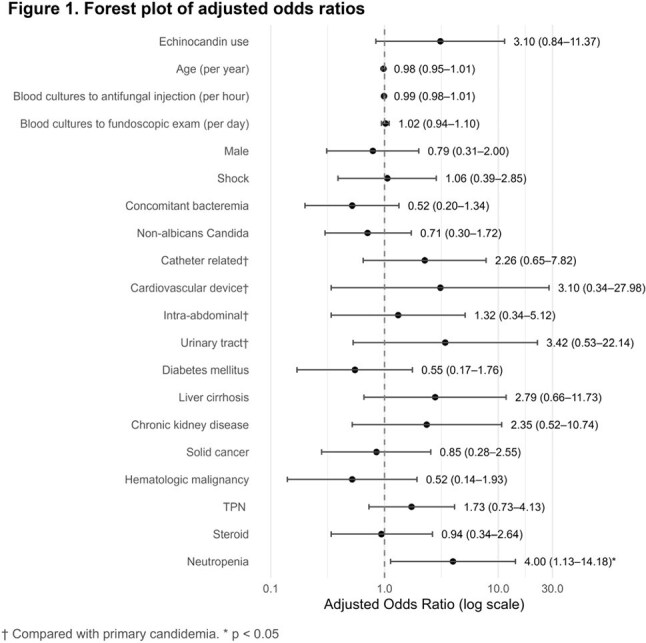

**Methods:**

We conducted a retrospective study at a 1,766-bed tertiary hospital in Seoul, Korea, including adults (≥19) with candidemia who underwent ophthalmologic exam within 30 days and received antifungal therapy. Exclusion criteria were prior antifungal use, combination therapy, < 3 days of treatment, antifungal resistance, or polymicrobial candidemia. OC was defined as chorioretinitis, vitritis, or nonspecific retinal lesions. Multivariable logistic regression was used to assess factors associated with OC. A subgroup analysis was performed in patients without neutropenia.

**Figure:**
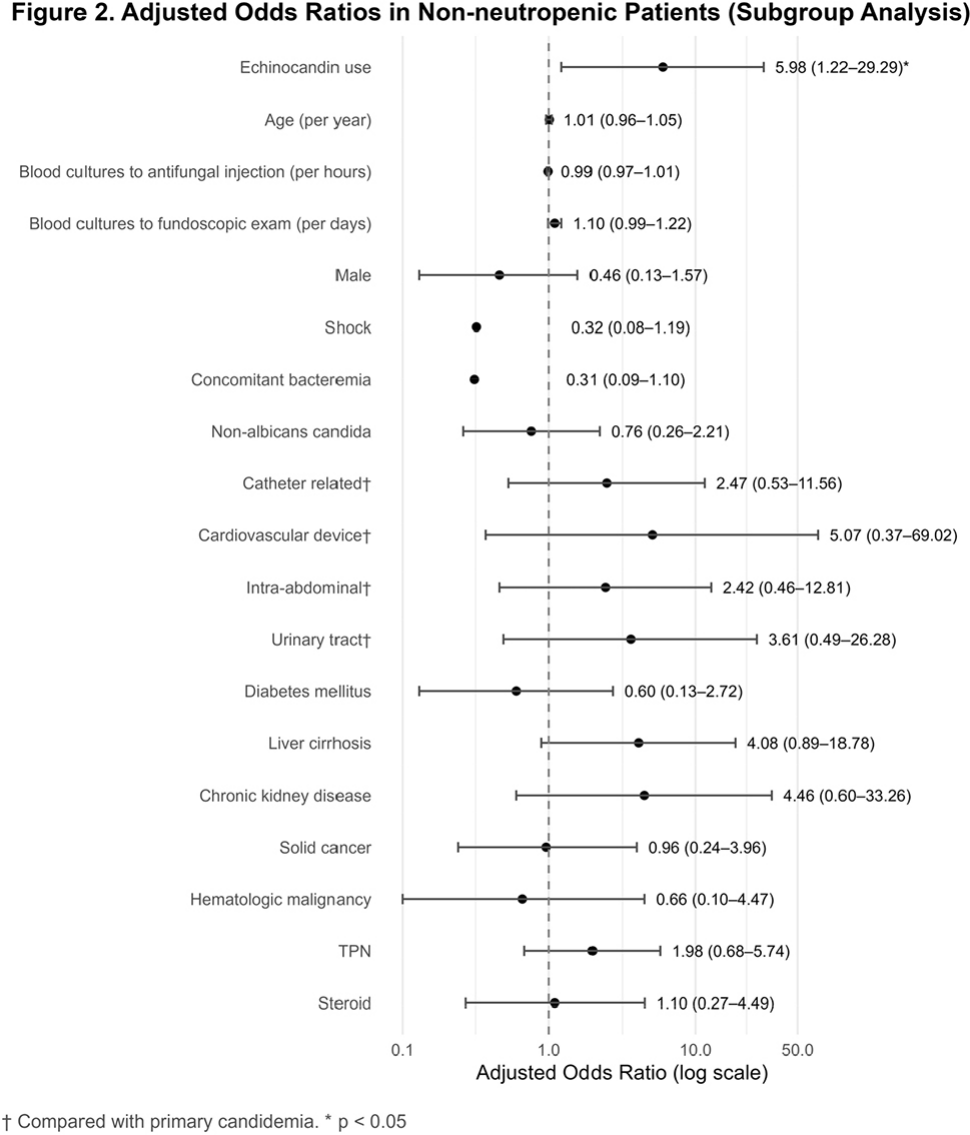

**Results:**

Among 137 patients, 30 received non-echinocandin and 107 received echinocandin as initial antifungal agents. Hematologic malignancy, neutropenia, and steroid use were more common in the echinocandin group. Intra-abdominal infections predominated in the non-echinocandin group; primary and catheter-related infections were more frequent in the echinocandin group. *C. albicans* was the most common species. In multivariable analysis, echinocandin use was not significantly associated with OC (aOR 3.10, 95% CI 0.84–11.37; p=0.088), but neutropenia was (aOR 4.00, 95% CI 1.13–14.18; p=0.032). In the non-neutropenic subgroup, echinocandin use was significantly associated with OC (aOR 5.98, 95% CI 1.22–29.29; p=0.027).

**Conclusion:**

Initial echinocandin use was not associated with increased OC risk overall. However, in non-neutropenic patients, echinocandin use was associated with higher OC risk, suggesting the need for caution when empirical therapy is initiated in this subgroup.

**Disclosures:**

All Authors: No reported disclosures

